# Multivalent Interactions of Human Primary Amine Oxidase with the V and C2_2_ Domains of Sialic Acid-Binding Immunoglobulin-Like Lectin-9 Regulate Its Binding and Amine Oxidase Activity

**DOI:** 10.1371/journal.pone.0166935

**Published:** 2016-11-28

**Authors:** Heli Elovaara, Vimal Parkash, Ruth Fair-Mäkelä, Outi M. H. Salo-Ahen, Gabriela Guédez, Eva Bligt-Lindén, Janne Grönholm, Sirpa Jalkanen, Tiina A. Salminen

**Affiliations:** 1 Medicity Research Laboratory, University of Turku, Turku, Finland; 2 Structural Bioinformatics Laboratory, Biochemistry, Faculty of Science and Engineering, Åbo Akademi University, Turku, Finland; 3 Turku Doctoral Program of Molecular Medicine, University of Turku, Turku, Finland; 4 Pharmaceutical Sciences Laboratory, Pharmacy, Faculty of Science and Engineering, Åbo Akademi University, Turku, Finland; 5 Department of Medical Microbiology and Immunology, University of Turku, Turku, Finland; Radboud university medical center, NETHERLANDS

## Abstract

Sialic acid-binding immunoglobulin-like lectin-9 (Siglec-9) on leukocyte surface is a counter-receptor for endothelial cell surface adhesin, human primary amine oxidase (hAOC3), a target protein for anti-inflammatory agents. This interaction can be used to detect inflammation and cancer *in vivo*, since the labeled peptides derived from the second C2 domain (C2_2_) of Siglec-9 specifically bind to the inflammation-inducible hAOC3. As limited knowledge on the interaction between Siglec-9 and hAOC3 has hampered both hAOC3-targeted drug design and *in vivo* imaging applications, we have now produced and purified the extracellular region of Siglec-9 (Siglec-9-EC) consisting of the V, C2_1_ and C2_2_ domains, modeled its 3D structure and characterized the hAOC3–Siglec-9 interactions using biophysical methods and activity/inhibition assays. Our results assign individual, previously unknown roles for the V and C2_2_ domains. The V domain is responsible for the unusually tight Siglec-9–hAOC3 interactions whereas the intact C2_2_ domain of Siglec-9 is required for modulating the enzymatic activity of hAOC3, crucial for the hAOC3-mediated leukocyte trafficking. By characterizing the Siglec-9-EC mutants, we could conclude that R120 in the V domain likely interacts with the terminal sialic acids of hAOC3 attached glycans whereas residues R284 and R290 in C2_2_ are involved in the interactions with the active site channel of hAOC3. Furthermore, the C2_2_ domain binding enhances the enzymatic activity of hAOC3 although the sialic acid-binding capacity of the V domain of Siglec-9 is abolished by the R120S mutation. To conclude, our results prove that the V and C2_2_ domains of Siglec-9-EC interact with hAOC3 in a multifaceted and unique way, forming both glycan-mediated and direct protein-protein interactions, respectively. The reported results on the mechanism of the Siglec-9–hAOC3 interaction are valuable for the development of hAOC3-targeted therapeutics and diagnostic tools.

## Introduction

Sialic acid-binding immunoglobulin-like lectins (Siglecs) are a family of proteins expressed on different haemopoietic and immune system cells [1, 2]. Based on their homology to CD33/Siglec-3, the CD33-related Siglecs form a subgroup of the Siglec family. In addition to Siglec-3, the subgroup includes Siglec-9, -5 to -11, -14 and -16 [3], which are able to bind to a variety of sialyl sugars and can regulate the immune response [3, 4]. Siglec-9 is an immunosuppressive molecule expressed mainly on neutrophils, monocytes, macrophages, as well as dendritic and NK-cells [2, 3]. It consists of three extracellular immunoglobulin-like domains: a V-set domain followed by two C2-set domains and a short cytosolic tail including the Immunoreceptor Tyrosine-based Inhibition Motif (ITIM) and ITIM-like motifs [5].

Siglec-9 is also a leukocyte trafficking molecule and its expression is rapidly up-regulated on the leukocyte surface after inflammation stimuli [6]. Recently, we have identified Siglec-9 and Siglec-10 as counter receptors for human primary amine oxidase (hAOC3; also called vascular adhesion protein-1, VAP-1) on the endothelial cell surface [6, 7]. Similar to Siglec-9, hAOC3 is an inflammation-inducible protein [8, 9]. Upon inflammation, leukocytes migrate from the blood into the non-lymphoid tissues and the heavily glycosylated hAOC3 contributes to several steps in the extravasation cascade and controls the trafficking of lymphocytes, granulocytes and monocytes to the sites of inflammation [10]. Besides being an adhesion molecule, hAOC3 is also an enzyme, which catalyzes oxidative deamination of primary amines and produces hydrogen peroxide, aldehyde and ammonium [11]. The catalytic site of hAOC3 is deeply buried and contains an essential topaquinone (TPQ) cofactor, modified from Tyr471 in a copper-dependent manner. The two functions of hAOC3 are interlinked since inhibition of the enzymatic activity of hAOC3 increases rolling velocity but reduces adhesion and transmigration steps of leukocyte extravasation *in vivo* [12]. Additionally, sialic acids of the hAOC3-attached glycans are crucial for adhesion [13] and the hAOC3 glycosylation is important in the initial recognition but also regulates the enzymatic activity [14]. Since the small molecular inhibitors of hAOC3 oxidase activity are shown to prevent the inflammatory function of hAOC3 *in vivo*, the hAOC3 inhibitors could be used in treating acute and chronic inflammatory conditions as well as tumor progression and metastatic spread of cancer (reviewed in [15]).

Siglec-9 and Siglec-10 were initially identified as potential ligands for hAOC3 using the CX8C phage peptide library [6, 7]. The current knowledge on the Siglec-9–hAOC3 interaction is mainly obtained from studies with peptides, which correspond to the CE loop of the second C2 (C2_2_) domain of Siglec-9 [6]. Based on the previous results, two arginines in the peptide were crucial for the interaction. These correspond to R284 and R290 in Siglec-9 and when only one of the arginines was present in the peptide, the binding to hAOC3 reduced while mutating both of them totally abolished binding. As hAOC3 is translocated to endothelial cell surface mainly upon inflammation and in certain cancers, Siglec-9 peptides are valuable as diagnostic tools to detect inflammation and cancer *in vivo*. In fact, the labeled Siglec-9 peptide functions as a tracer in the positron emission tomography (PET) of hAOC3. [6].

In the present study, we have expressed and purified the extracellular region of Siglec-9 (Siglec-9-EC) to investigate its interaction with hAOC3 at the protein level. Firstly, we used mutagenesis to find out the individual effects of R284 and R290 in the C2_2_ domain and, secondly, we inspected if the V domain of Siglec-9 also plays a role in the interaction. Since R120 in the V domain of Siglec-9 has been reported to be crucial for the recognition of μ2–3 and μ2–6-linked sialic acids on some other Siglec-9 ligands [16], we created the ΔV and R120S mutants of Siglec-9 to study the interaction of the V domain with the hAOC3-attached sialoglycans. Biochemical studies of the Siglec-9-EC interaction with hAOC3, supported by mutagenesis and structural modeling, allowed us to show that the V domain of Siglec-9 binds the sialic acids on the hAOC3 surface and the C2_2_ domain of Siglec-9 interacts with the active site channel of hAOC3. Our results provide novel insights into the mechanism of the physiologically relevant interactions that occur between leukocytes and endothelial cells upon inflammatory conditions and, thus, aid the further development of hAOC3-targeted therapeutics and diagnostic tools.

## Materials and Methods

### Comparative 3D modeling

**Fig 1:**
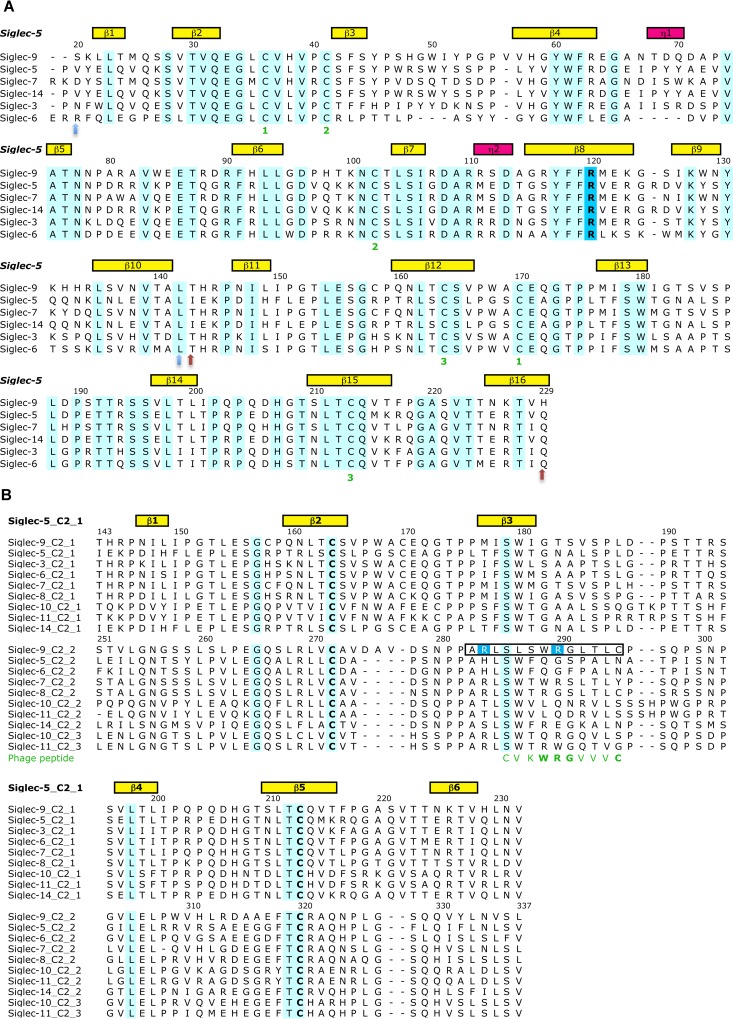
The multiple sequence alignments used in the 3D modeling of Siglec-9-EC. The sequence numbering (according to the Siglec-9 sequence) and the secondary structural elements (yellow and pink boxes denoting the beta sheets and the 3/10-alpha helices, respectively) in the Siglec-5 structure (PDB ID 2ZG2), which was used as a template in the modeling procedure, are shown above both of the alignments. The conserved residues have a cyan background. **(A)** Multiple sequence alignment used in the 3D modeling of the V-C2_1_ domains of Siglec-9. In the sequence alignment of the V-C2_1_ domains of Siglec-3, -5, -6, -7, -9 and -14, the six cysteine residues forming disulphide bonds are marked as ‘1’, ‘2’ and ‘3’ (in green) below the alignment. The key sialic acid binding residue, R120, is highlighted in blue background. The two light blue arrows below the alignment mark the V domain and the light brown arrows define the C2_1_ domain. **(B)** Multiple sequence alignment of the C2 domains of CD-33-related Siglec sequences used in the 3D modeling of the C2_2_ domain of Siglec-9. The sequence alignment includes the C2_1_, C2_2_ and C2_3_ domains. The key arginine residues, R284 and R290, are highlighted with a blue background. The phage peptide sequence is shown in green under the sequence alignment. The Siglec-9 peptide sequence used in the PET study [6] is boxed in the alignment. The conserved cysteine residues forming the disulphide bond within the C2 domain are in bold letters.

The 3D structural model for Siglec-9-EC sequence (UniProt Knowledgebase (UniProtKB) Q9Y336) was constructed using the X-ray structure of Siglec-5 (Protein Data Bank Identification Code (PDB ID) 2ZG2 [17]) as a template. Firstly, a 3D model for the V–C2_1_ domains (residues S20-H229) was made based on the multiple sequence alignment of Siglec-3, -5, -6, -7, -9 and -14 using the V–C2_1_ domains of the Siglec-5 crystal structure as a template (Fig 1A). Secondly, the C2_2_ domain (residues S251-L335) was separately modeled using the sequence alignment of the C2 domains of CD-33-related Siglec sequences, including all C2_1_, C2_2_ and C2_3_ domains, and the C2_1_ domain of the Siglec-5 crystal structure as a template (Fig 1B). Thereafter, the linker region (L230-V250) between the C2_1_ and C2_2_ domains was modeled in two different ways. The first complete Siglec-9-EC model of residues S20-L335 (model 1) was done by orienting the domains manually into an extended 3D arrangement and the second model of the same residues (model 2) was based on the 3D arrangement of the immunoglobulin domains in the X-ray structure of neural cell adhesion protein (PDB ID 1QZ1 [18]), which also has an extended 3D arrangement of the immunoglobulin domains. All sequence alignments were done using Malign [19] within Bodil [20]. For all the 3D models, ten models were generated with Modeller [21] and the ones with the lowest objective function were chosen as a representative for each model. The quality of the 3D folds of the V–C2_1_ and C2_2_ homology models were evaluated with PROCHECK [22], ProSA web [23, 24] and QMEAN [25]. To visualize the location of the sialic acid (SA) binding site in Siglec-9-EC, sialic acid binding mode was modeled based on the Siglec-5 complex (PDB ID 2ZG1 [17]). Model 1 and model 2 of Siglec-9-EC were refined by molecular dynamics (MD) simulations as described in the following section.

### Molecular dynamics simulations

Protein Preparation Wizard, as implemented in Maestro v. 9.6 molecular modeling software (Schrödinger, Inc.), was used to prepare model 1 and model 2 of Siglec-9-EC for the MD simulations. All hydrogen atoms were added, bond orders were assigned, and disulphide bridges were created between the following cysteine residues: 36 and 170, 41 and 102, 164 and 213, and 271 and 320. Hydrogen bonds were assigned at pH 7.0 and the protonation states of histidines were selected interactively to optimize the hydrogen bond network. Finally, a restrained energy minimization of hydrogen atoms was run in the OPLS 2005 force field.

Energy minimization, thermal equilibration and standard production simulations were performed with the AMBER package (version 12) [26] using the AMBER ff03 force field [27]. All simulations were run in an octahedral box (extending 10.0 Å from the protein), filled with explicit TIP3P water molecules [28] and one neutralizing Cl-ion. Periodic boundary conditions, particle-mesh Ewald electrostatics [29] and a cut-off of 9 Å for non-bonded interactions were used. A time step of 1 fs (for Langevin dynamics during equilibration) or 2 fs was applied together with the SHAKE algorithm [30] to constrain the bonds to hydrogen atoms. The 20-ns production simulations were performed at a constant temperature of 300 K and a pressure of 1 bar. The coupling constants for temperature and pressure [31] were 5.0 and 2.0 ps, respectively. Energy minimization was performed with the steepest descent and conjugate gradient methods in six steps, gradually reducing the restraints on the protein atoms to their initial positions. At each step, the restraint force constant was defined as follows: 10, 5, 1, 0.1, 0.01 and 0 kcal/molÅ^2^. Each minimization step was carried out for a maximum of 200 iterations (of which the 10 first iterations were with the steepest descent method and the rest with the conjugate gradient algorithm). Equilibration simulations were performed in five steps: (i) 10 ps heating of the system from 10 K to 300 K using a Langevin thermostat with a collision frequency (γ) of 1.0 ps^-1^, constant volume, and restraints on the protein atom positions (restraint force constant of 5 kcal/molÅ^2^); (ii) same as the previous step but for 20 ps and without restraints on the protein atom positions; (iii) 20 ps MD at 300 K using a Langevin thermostat with γ = 0.5 ps^-1^ and constant volume, no restraints on the protein; (iv) 50 ps MD at 300 K using a Langevin thermostat with γ = 0.5 ps^-1^ and constant pressure of 1.0 bar, coupling constant for pressure of 1.0 ps, no restraints on the protein. (v) 400 ps MD at 300 K and at constant pressure of 1 bar, coupling constants for temperature and pressure were 5.0 and 2.0 ps, respectively, no restraints on the protein. The MD simulation trajectories were analyzed with VMD [32] and the ptraj module of AMBER. The resulting final frame structures were first minimized with AMBER (similarly to the last step of the initial minimization) and then visually examined with PyMOL (Schrödinger, Inc.).

### Reagents

All reagents, if not otherwise mentioned, were purchased from Sigma-Aldrich.

### Antibodies

To monitor the transfection efficiency or protein surface expression, we used immunofluorescence staining and monoclonal antibodies TK-8-14 (hAOC3) and Kalli (Siglec-9) as well as polyclonal antibody (Siglec-9, R&D) [5, 33]. A negative control antibody in the immunofluorescence staining was NS-1 (mouse IgG1 ATCC TIB-18).

### Vectors and cell lines

For the insect cell production of Siglec-9, we inserted a HindIII restriction endonuclease site after the carboxyl terminal His-tag by inverse PCR (The primer sequences are shown in S1 Text). The PCR product was then cut with XbaI and HindIII and inserted into pFastBac1 derivative vector p503.9 [34]. To include a cleavable His-tag, we changed the tag from C-terminus to N-terminus by PCR. The resulting constructs had then the secretion signal for insect cell production, N-terminal flag and His-tags and coding region for Siglec-9-EC (V-C2_12_; residues 26–348) and Siglec-9-ΔV (C2_12;_ residues 145–348). The R284S, R290S, R120S, R120S/R284S and R120S/R290S mutations were produced using QuickChange Lightning–mutagenesis kit (Agilent Technologies). For the PCR we used a Siglec-9-EC production vector as a template, primers HE-56-61 (The primer sequence, S1 Text) and followed the manufacturer’s instructions exactly. The mutations were confirmed by sequencing the whole Siglec-9 gene.

*Spodoptera frugiperda* (Sf9) cells and *Trichoplusia* ni (Tn5) cells were used for the expression and production of Siglec-9. The stable Chinese hamster ovary (CHO) cell lines CHO-hAOC3 and CHO-Siglec-9 have been described [5, 35]. To assess the transfection efficiency of CHO-Siglec-9, we did immunofluorescence staining using Kalli monoclonal antibody against Siglec-9 (see Antibodies).

### Production and purification of recombinant Siglec-9

Using Sf9 cells, high titer baculovirus stocks for each Siglec-9-EC construct were generated. For protein expression, the High Five Tn5 cells were infected with the baculovirus stock. Two days post infection, the protein was secreted out into the medium, and the supernatant was harvested by centrifugation to remove cellular material. The 6×His tagged protein was purified by adding Ni^2+^-resin (Ni^2+^-charged chelating sepharose, GE Healthcare) to the supernatant in batch. After 45 minutes incubation at +4°C, the resin was washed with phosphate buffered saline (PBS) in the presence of 7 mM imidazole and the protein was eluted with 500 mM imidazole in PBS, pH 8.0. Finally, the protein was purified by gel filtration on a Superdex 200 10/300 GL (GE Healthcare) column in 20 mM HEPES pH 7.4, 150 mM NaCl. hAOC3 production and purification was carried out exactly as previously described [36].

### Thermal stability assays

The purified proteins were characterized using fluorescence based thermal stability assay [37]. In this methodology, a dye intercalates with the exposed hydrophobic regions generated by unfolding of proteins. We used SYPRO^®^ orange dye with maximal absorption of dye-protein complex at 470 nm and maximal emission at 569 nm. Siglec-9-EC was concentrated to 2 mg/ml. A 96-well plate was filled with protein samples, buffers and dye with assay volume of 25 μl per well. The analysis was done on an *iCycler* machine (Bio Rad Laboratories) and melting curves were generated by increasing the temperature from 20°C to 95°C with a stepwise increment of 1°C. The fluorescent signal is plotted as a function of temperature, and the significant increase in the signal (slope) corresponds to the melting of the protein. The analysis of the results and the melting temperature (T_m_) estimation of thermal shift assay were calculated with the Meltdown program [38]. The program estimates the melting temperature in two ways: by using a quadratic fit to the data around the global minimum of the first derivative curve (this value is used as the T_m_ in subsequent analyses) and by finding the temperature associated with the midpoint in the fluorescence response between the high point and the low point of the melt curve. The melt curves are considered to be normal by the Meltdown program if the estimated T_m_ values are within 5°C.

### Binding assay using surface plasmon resonance (SPR)

The binding of purified Siglec-9-EC to immobilized hAOC3 was measured with BiacoreX (GE Healthcare). AOC3 was expressed in the CHO cells and purified as described in Smith et al. (1998) [11]. The preparation of the hAOC3 chip was done via amine coupling according to manufacturer’s instructions, using 10 mM Na-acetate buffer pH 4.0 as a coupling buffer, resulting to 10 000 RU of hAOC3 immobilized on a CM5 chip. To monitor the binding of Siglec-fragment to hAOC3, we injected 20 μl of 0.3–31 μM of Siglec-9-EC over the surface at 25°C and recorded the response units (RU) as a function of time. The running buffer was HEPES buffered saline (HBS, 10 mM HEPES, 150 mM NaCl, pH 7.4) with 0.005% Surfactant-P20 (GE Healthcare). 25 mM HBS with or without 5% glycerol was used as a protein buffer and the dilutions were made in the purification buffer. During the measurement, the binding to an empty reference channel was subtracted. We also monitored the response of the protein buffer alone. Two experiments were done using different protein preparations. To determine a binding constant for Siglec-9-EC, we plotted all the responses as a function of concentration and used non-linear regression of GraphPad4 software (GraphPad Software Inc., La Jolla, CA).

To test if mutations of R284 and R290 in Siglec-9 have any effect on the binding to hAOC3, we monitored the binding of Siglec-9-EC and the mutant proteins Siglec-9-EC/R284S and Siglec-9-EC/R290S on immobilized hAOC3 using from five to seven different concentrations. We then determined *k*_*off*_ and *k*_*on*_ separately using the Biaeval program, and determined the binding constants for every curve separately. For the final *K*_*d*_, we did not include the highest and lowest value when we calculated the average values. Because Siglec-9-EC-R120S, -R120S/R284S, -R120S/R290S and–ΔV bound very weakly to hAOC3, it was not possible to determine their binding constants and, thus, only one concentration (0.5 μM) of these proteins was tested.

The effect on Siglec-9-EC–hAOC3 interaction was also tested by SPR for two different glycans, sialic acid and disialyl lactotetraosylceramide (DSLc4), and for an imidazole molecule known to block access into the hAOC3 active site. In order to monitor the effect of a particular molecule, we incubated Siglec-9-EC in the purification buffer with different concentrations of above mentioned reagents on ice for minimum of 30 min before the measurement. After the incubation, Siglec-9-EC with the reagent was injected as above. We also tested if semicarbazide, a known inhibitor binding irreversibly to the topaquinone cofactor of hAOC3, has an effect on the interaction between Siglec-9 and hAOC3. Before Siglec-9-EC injections, we injected 1 mM semicarbazide over the hAOC3 surface to bind it to hAOC3 active sites. After this, we monitored the binding of Siglec-9-EC to hAOC3 and included 1 mM semicarbazide also in the injected Siglec-9-EC solutions. For every measurement the relative binding was calculated by normalizing the responses to the first control condition, which was set to 1.0.

### Siglec-9 as a hAOC3 substrate

The enzymatic activity of hAOC3 on purified Siglec-9-EC was assayed as described earlier in e.g. [36]. Now we used 25 mM HEPES pH 7.4, 150 mM NaCl as a reaction buffer and 30 μg of CHO-hAOC3 lysate as the protein source, to determine the specific activity for hAOC3. As a positive control we used 0.25 mM benzylamine as a substrate and as a negative control the lysate of CHO-hAOC3-Y471F cells expressing an inactive hAOC3 mutant [12]. The formation of fluorescence was followed for 1–3 hours. For every experiment we used duplicate wells.

### Siglec-9 as a modulator of hAOC3 activity

To find out if Siglec-9 has an influence on the amine oxidase activity of hAOC3, we performed the activity assay with live CHO-hAOC3 cells and used labeled benzylamine as a substrate as previously described [10, 39, 40]. In detail, we plated 5x10^4^ cells per well on a 96-well plate a day before the experiment, and cultured the cells in 200 μl of F-12 medium (Gibco). Before the experiment, we removed the medium and added first the reaction buffer (20 mM HEPES, 5 mM KH_2_PO_4_, 1 mM MgSO_4_, 1 mM CaCl_2_, 136 mM NaCl, and 4.7 mM KCl, pH 7.4) and 1 mM clorgylin and, thereafter, 1 mM semicarbazide inhibitor or 5 μM Siglec-9-EC or bovine serum albumin (BSA). The assay of the R120S, R120S/R284S and R120S/R290S Siglec-9-EC mutant proteins was done with 1 μM protein concentration. The cells were then incubated at 37°C/5% CO_2_ for 20 min, after which [7-^14^C]benzylamine (Amersham Pharmacia, 54 mCi/mmol) was added (2.5 μM). The cells were incubated for further 2 hours at 37°C/5%CO_2_ after which the reaction was stopped with 2 M citric acid and the labeled reaction product benzaldehyde was extracted into toluene for the liquid scintillation counting (Wallac-1409 Liquid scintillator, Wallac, Turku, Finland). Three independent experiments with at least duplicates were performed and the relative activities were calculated by normalizing the responses to the first positive control condition.

### Statistics

We used non-parametric Mann-Whitney U-test for the comparison of means. Analysis of variance was done using Kruskall-Wallis test. The p-values below 0.05 were considered significant. All the analyses were done using IBM SPSS Statistics version 22.0 (SPSS Inc. USA).

## Results

### Characterization of recombinant Siglec-9-EC

#### Production and purification of recombinant Siglec-9-EC

**Fig 2:**
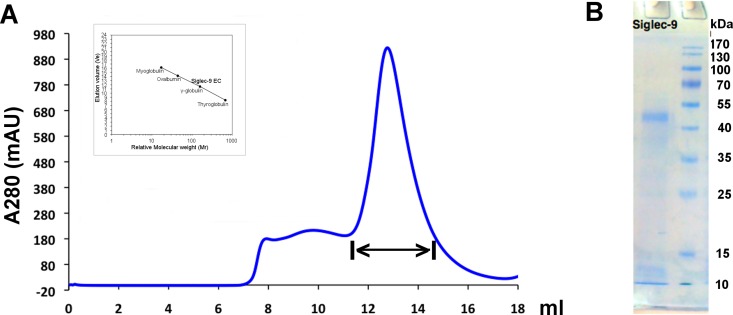
Purification of Siglec-9-EC from the culture medium. **(A)** Size exclusion chromatogram of the final purification step for Siglec-9-EC. The fractions from the peak region, marked with double arrow, were collected. The Siglec-9-EC mutants were purified using the same protocol and gave similar curves (data not shown). The calibration curve of the standard proteins, bovine thyroglobulin (670 kDa), bovine γ-globulin (158 kDa), chicken ovalbumin (44 kDa) and horse myoglobulin (17 kDa), is shown aside the chromatogram. **(B)** SDS-PAGE analysis of the purified wild-type (WT) sample that was collected from the peak region shown in Fig 2A.

We have previously shown that Siglec-9 peptides bind to hAOC3 and that CHO-Siglec-9 cells interact with CHO-hAOC3 cells. Further, we have demonstrated *ex vivo* that Siglec-9 mediates the binding of human granulocytes to hAOC3 expressed on lymph node vasculature of transgenic mice [6]. To analyze the biologically relevant hAOC3–Siglec-9 interactions in more detail at the protein level, we now expressed the extra-cellular part of Siglec-9 (Siglec-9-EC) as a recombinant protein in Tn5 insect cells. The protein was purified from the culture medium by Ni-affinity chromatography, followed by size exclusion chromatography (Fig 2A). The purity of Siglec-9-EC on the SDS-PAGE was estimated to be more than 95% (Fig 2B). Based on the visual inspection of the SDS-PAGE, the size of the denatured Siglec-9-EC is around 50 kDa, which is higher than the 40 kDa molecular weight calculated for Siglec-9-EC with tags. Using the retention time calculation based on the chromatogram (Fig 2A), the native Siglec-9-EC is a dimer of 85 kDa. This measurement gives a molecular weight of 42.5 kDa for the monomer, which is also slightly larger than the estimated size. The observed larger molecular weight of Siglec-9-EC might result from the fact that Siglec-9-EC has eight putative N-glycosylation sites (UniProtKB Q9Y336) and glycosylation might increase its size. Dimerization of Siglec has also been observed for Siglec-5 and Siglec-8 [41, 42].

As R284 and R290 in the C2_2_ domain of Siglec-9 were proposed to interact with hAOC3 [6], we created the R284S and R290S point mutations of Siglec-9-EC to find out if these residues indeed have a role in the Siglec-9-EC–hAOC3 interaction. To study whether the V domain of Siglec-9 is also involved in the interactions, we first deleted the sialic acid binding V domain (Siglec-9-ΔV) and thereafter created the R120S mutant to abolish the sialic acid binding capability of the V domain. Unlike Siglec-9-ΔV, which totally lacks the V domain, Siglec-9-EC/R120S variant retains the domains of Siglec-9-EC intact. Additionally, we created R120S/R284S and R120S/R290S double-mutants. The purity of the mutant proteins was similar to that of the WT protein (data not shown). All Siglec-9 proteins gave about 1 mg of purified protein per liter of the culture volume.

#### Stability analysis of the produced Siglec-9-EC proteins with thermal shift assay

**Fig 3:**
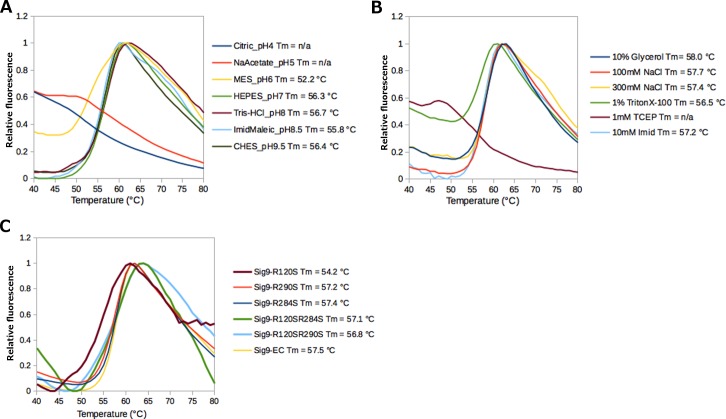
Characterization of Siglec-9-EC with the fluorescence-based thermal shift assay. **(A)** The effect of different buffer pH values on the stability of Siglec-9-EC. The tested buffers were: Citric_pH4 = 100 mM citrate buffer pH 4.0, NaAcetate_pH5 = 100 mM Sodium acetate buffer pH 5.0, MES_pH6 = 100 mM MES buffer pH 6.0, HEPES_pH7 = 100 mM HEPES buffer pH 7.0, Tris-HCl_pH8 = 100 mM Tris-HCl buffer pH 8.0, ImidMaleic_pH 8.5 = 100 mM N-imidazolyl maleamic acid pH 8.5, CHES_pH 9.5 = 100 mM CHES buffer pH 9.5. All of them contained 125 mM NaCl. **(B)** Thermal shift assay of Siglec-9-EC in 20 mM HEPES buffer, 150mM NaCl, pH 7.4 in the presence of different additives. **(C)** Thermal shift assay of Siglec-9-EC and the mutant Siglec-9-EC proteins to check the effect of the mutations on the stability of the protein.

To assess the folding of Siglec-9-EC produced in insect cells, we performed fluorescence-based thermal shift assay using *iCycler*. This methodology takes advantage of the fact that the SYPRO orange dye becomes fluorescent when it binds to hydrophobic amino acids in the protein and, thus, when the protein starts to unfold during heat denaturation, the hydrophobicity of the dye environment increases and can be detected as an increased fluorescence. The results were analyzed and the melting temperature (T_m_) was calculated using the Meltdown program [38]. First, we used a buffer screen, which contains a set of seven different buffer systems each at a concentration of 100 mM covering a pH range from 4.0 to 9.5 in the presence of 125 mM NaCl (Fig 3B). On the basis of thermal shift measurements, Siglec-9-EC seems to be stable in most of the buffer conditions at different pH values (up to pH 9.5), except for pH 6.0 and below (Fig 3A). Furthermore, the thermal stability of Siglec-9-EC was studied in the presence of different additives. The results suggest that addition of the reducing agent 1 mM TCEP and detergent 1% Triton X-100 affects the protein stability and slightly reduces the T_m_ of the protein (Fig 3B). Based on these measurements, we concluded that the purified Siglec-9-EC is most stable in 20 mM HEPES, 150 mM NaCl, pH 7.4, which was therefore selected as the storage buffer for the proteins. Finally, we ran the thermal shift assay for the purified Siglec-9-EC/R284S, Siglec-9-EC/R290S, Siglec-9-EC/R120S, Siglec-9-EC/R120S/R284S and Siglec-9-EC R120S/R290S mutant samples, which all showed similar melting curves and no change in the T_m_ of 57°C, except for the slightly lower T_m_ of 54.2°C observed for Siglec-9-EC/R120S (Fig 3C). This suggests that the arginine to serine mutations in Siglec-9-EC did not have an effect on its fold and stability and, thus, proved that our strategy of avoiding hydrophobic patches on the surface of Siglec-9-EC by replacing the positively charged arginines with polar serines instead of hydrophobic alanines was successful.

### The 3D model for Siglec-9-EC reveals the position of the key arginines involved in its interaction with hAOC3

The 3D structural model for Siglec-9-EC consisting of domains V-C2_1_-C2_2_ was created to illustrate the location of the two arginine residues, R284 and R290 (in the C2_2_ domain), which we have earlier found to be critical for the binding of Siglec-9-derived peptides into hAOC3 [6]. We also wanted to correlate their position to that of R120 (in the V domain), the key residue for the sialic acid binding. Firstly, the 3D model for the V-C2_1_ was generated by homology modeling based on the multiple sequence alignment (Fig 1A) and the corresponding domains of the Siglec-5 X-ray structure used as a structural template for modeling (PDB ID 2ZG). Secondly, the 3D model for the C2_2_ domain was created based on the alignment of the C2 domains of several Siglecs (Fig 1B) using the crystal structure of the C2_1_ domain of Siglec-5 with the C2 fold as a structural template. The V-C2_1_ and C2_2_ models were visually inspected and compared with the 3D structure of Siglec-5, and their quality was assessed with several programs/servers that all gave acceptable results. According to PROCHECK [22], 91.8% of the V-C2_1_ residues and 94.7% of the C2_2_ residues are in the favored regions of the Ramachandran plot. Thus, the stereochemical quality of the models is actually better than that of the Siglec-5 structure (the corresponding value is 86.2%). Analysis of the homology models with the ProSAweb [23, 24] gave Z-scores of -5.9 for V-C2_1_ (Figure A in S1 Fig) and -3.87 for C2_2_, (Figure B in S1 Fig) with the average energy over a 40 amino acid window below the threshold), which are within the range of the scores typically found for experimentally determined structures of similar size. The QMEAN Z-scores [25] of the 3D models were -1.91 for V-C2_1_ (Figure C in S1 Fig) and -1.07 for C2_2_ (Figure D in S1 Fig), which are within the range of the scores for the set of non-redundant PDB structures of the same size and reflect the overall reliability and good quality of the models.

**Fig 4:**
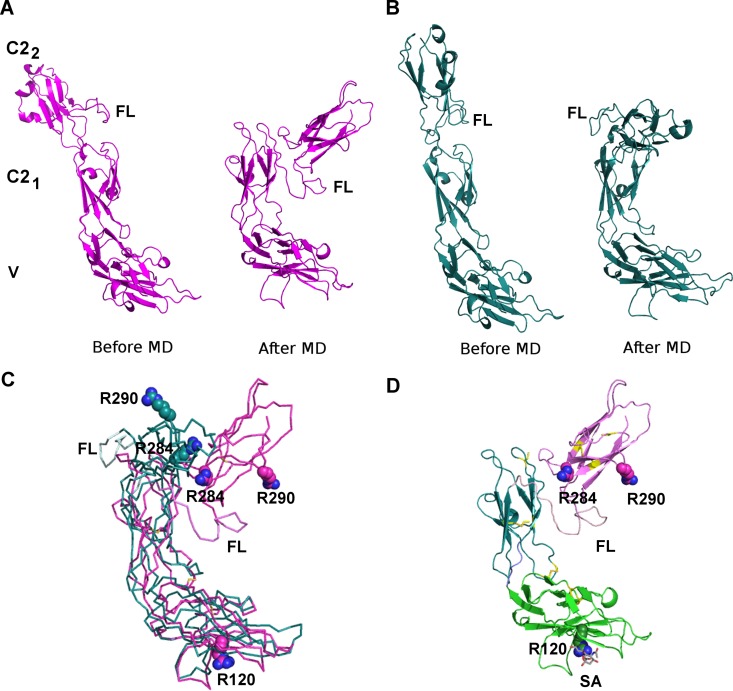
The 3D model for Siglec-9-EC. The two alternative models for Siglec-9-EC with a differently modeled flexible linker region (FL, between C2_1_ and C2_2_ domains) before and after the refinement by MD simulations. **(A)** Model 1. **(B)** Model 2. **(C)** Superimposition of the V-C2_1_ domains of the two models shows a slightly different orientation for the C2_2_ domain. Model 1 in magenta and model 2 in cyan. **(D)** The best model for Siglec-9-EC (model 1). Sialic acid (SA; in grey sticks) is modeled to interact with R120 in the V domain. The V domain is shown in green, C2_1_ in blue and C2_2_ in magenta. In **(C)** and **(D)**, R120 in the V domain and R284 and R290 in the C2_2_ domain are shown as spheres and cysteines forming the disulphide bridges in yellow.

The linker region (L230-V250) between the C2_1_ and C2_2_ domains is predicted to be highly flexible and its position has a significant effect on the relative orientation of the V and C2_2_ domains. Thus, the linker region (L230-V250) was modeled by manually creating an extended 3D arrangement of the domains (Fig 4A, model 1) and by using the spatial 3D arrangement of the three immunoglobulin domains in the X-ray structure of the neural cell adhesion molecule (PDB ID 1QZ1 [18]; Fig 4B, model 2), which has a similar extended conformation. To refine the structures of the Siglec-9-EC models, both models were subjected to a 20-ns MD simulation, which resulted in similar, bent conformations (Fig 4A–4C). Since model 1 was energetically better (prior to and after MD), it was used as a representative model for the further analysis (Fig 4D).

The model for the V-C2_1_ domain is the most reliable part of the final model due to the relatively high sequence identity (~50%) between these domains in Siglec-9 and Siglec-5. Furthermore, a conserved disulphide bridge between Cys35 and Cys170 stabilizes the spatial orientation of the V and C2_1_ domains. As a result of the interdomain disulphide bridge the relative orientation of the V-C2_1_ domains is the same in the extended and bent conformation (Fig 4A–4C) whereas the movement of the C2_2_ domain relative to V-C2_1_ is not restricted and C2_2_ moves closer to the V domain in the bent conformation (Fig 4C).

The positions of R284 and R290 in the C2_2_ domain are based on the 3D fold of the C2_1_ domain of Siglec-5 since there are no X-ray structures for the C2_2_ domain of Siglecs. Despite the low sequence identity between C2_2_ of Siglec-9 and C2_1_ of Siglec-5 (14%), the multiple sequence alignment (Fig 2B) shows six totally conserved residues and a conserved hydrophobicity profile. Moreover, the minor variations in the sequence lengths occur in the loop regions. Two of the totally conserved residues are cysteines that form a disulphide bond stabilizing the 3D fold of the C2 domain. In the C2_2_ of Siglec-9, the conserved internal disulphide bridge between residues C272 and C320 increases the reliability for the 3D positions of R284 and R290. Both R284 and R290 are exposed to solvent and located nearby each other in the CE loop region of the C2_2_ domain of the Siglec-9-EC model (Fig 4D). In the extended conformation (prior to MD; Fig 4A), they are far away from the V domain but in the bent conformation they come closer to R120 in the sialic acid binding site of the V domain (Fig 4D).

### Role of R284 and R290 in the Siglec-9-EC binding to hAOC3 under flow condition

**Fig 5:**
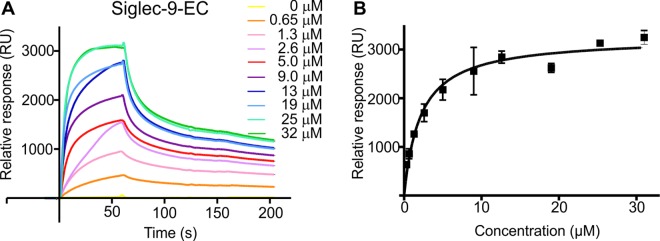
Recombinant Siglec-9-EC binds specifically to immobilized hAOC3. **(A)** The sensorgrams of binding of Siglec-9-EC to hAOC3 by SPR and **(B)** the curve regression of equilibrium responses in the function of concentration to determine apparent *K*_*d*_ by GraphPad (*n* = 2).

We next tested the binding of Siglec-9-EC on immobilized hAOC3 to determine the affinity between these proteins. Using SPR, we demonstrated that purified Siglec-9-EC bound specifically to hAOC3 with the affinity of *K*_*d*_ = 4.6 ± 0.93 μM and *B*_*MA*X_ = 3504 ± 204 RU, when we determined the apparent *K*_*d*_ by using the semi-quantitative method assuming a steady-state equilibrium at the end of the injection and a one-to-one binding model (Fig 5A and 5B).

Since we have earlier shown that the mutation of R284 and R290 in the Siglec-9–like peptides reduces the binding to hAOC3 [6], we now tested the binding of R284S and R290S mutants to the immobilized hAOC3. The binding constants (Table 1) show that neither of the Arg/Ser mutations abolished the binding, and when using the Biaeval program for determination of *k*_*on*_ and *k*_*off*_ and calculating *K*_*d*_ as a ratio of these, *K*_*d*_ was lower than with the semi-quantitative method (1.04 vs. 4.6 μM). When R284 was mutated to a serine, *K*_*d*_ improved about tenfold (0.14 ± 0.58 μM) whereas the R290S mutation had a smaller effect resulting to a *K*_*d*_ of 0.53 ± 0.50 μM. Although the error range of the constants is large, both of the Arg/Ser mutations caused a tighter binding. The maximal binding at highest concentrations of both mutants were lower (1130 RU for R284S, 1760 RU for R290S) than for the wild-type (WT) Siglec-9-EC (3120 RU) due to the lower concentrations used (Figure A in S2 Fig).

**Table 1:** Binding constants of Siglec-9-EC proteins on hAOC3. *K*_*d*_ of Siglec-9-EC/R284S differed significantly from *K*_*d*_ of Siglec-9-EC (Z = -2.61, *p* = .027), but *K*_*d*_(Siglec-9-EC/R290S) did not (Z = -1.57, *p* = .302).

Protein	*K*_*d*_ (μM)	*k*_*on*_ (10^4^ 1/Ms)	*k*_*off*_ (10^−3^ 1/s)
Siglec-9-EC	1.04±0.87	1.12±1.00	6.91±0.26
Siglec-9-EC/R284S	0.14±0.58	3.21±1.67	3.73±0.36
Siglec-9-EC/R290S	0.53±0.50	1.10±1.31	3.10±0.12

When separate *k*_*on*_ and *k*_*off*_ were compared between the WT and the Arg/Ser mutants, we noticed that the R284S mutant had three times higher *k*_*on*_ than the WT and a *k*_*off*_ about half of the WT (Table 1). Therefore, both increased association and decreased dissociation contribute to the significantly increased binding of R284S. On the contrary, the R290S mutation had an almost identical association rate as the WT but the decreased dissociation rate (Table 1) leads to better binding.

### The intact V-domain is crucial for the Siglec-9-EC–hAOC3 interaction

**Fig 6:**
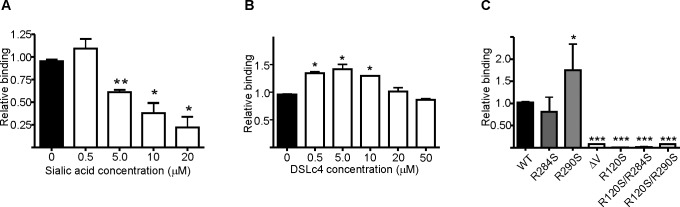
The role of sugars, sialic acid binding domain (ΔV) and the mutations R284S, R290S on the binding of Siglec-9-EC to hAOC3. **(A)** Sialic acid addition decreases the binding of Siglec-9-EC (WT) to hAOC3 by SPR. **(B)** The effect of the disialyl lactotetraosylceramide (DSLc4) sugar on hAOC3–Siglec-9-EC interaction. Each experiment was performed as duplicate. **(C)** Relative binding of Siglec-9-EC (WT), -R284S, -R290S, -ΔV, -R120S, R120S/R284 and–R120S/R290S mutants at concentration of 0.5 μM to immobilized hAOC3 (means of two (R120S based mutants)-three experiments are shown ± SEM). *: p<0.05, **:p<0.01 and ***:p<0.001.

As both **α**2,3- and **α**2,6-linked sialic acids of hAOC3 are known to be involved in cell adhesion [13], we tested if the V domain in Siglec-9 interacts with them. In this experiment, we first pre-incubated Siglec-9-EC with sialic acid and then assayed the binding to hAOC3 with SPR. We observed a clear dose-dependent inhibition in the binding of Siglec-9-EC to hAOC3 by sialic acid (Fig 6A, Figure B in S2 Fig).

We also tested the effect of a glycan disialyl lactotetraosylceramide (DSLc4) on the binding of Siglec-9-EC to hAOC3 using SPR. DSLc4 binds to Siglec-7 and aids dimer formation by binding to two Siglec-7 molecules [43] but it does not interact with Siglec-9 [44] and thus functions as a negative control for the binding assay. As expected, DSLc4 did not have an effect on the interaction at 20 and 50 μM concentrations (Fig 6B, Figure B in S2 Fig).

Because the sialic acid decreased the interaction of Siglec-9 with hAOC3, we next deleted the sialic acid binding V domain. When we assayed the relative binding of Siglec-9-ΔV on immobilized hAOC3, the binding of Siglec-9-ΔV remained at the background level (Fig 6C, Figure C in S2 Fig). Due to the low binding to hAOC3 (<10% of WT binding), we were not able to determine an accurate binding constant for Siglec-9-ΔV. To confirm that the sialic binding ability of Siglec-9 is mainly responsible for the binding of Siglec-9 to hAOC3, we mutated R120, the crucial sialic acid binding residue [16], to a serine and assayed the binding to hAOC3 (Fig 6C). Similar to Siglec-9-ΔV, the binding of Siglec-9-EC/R120S as well as the double mutants (R120S/R284S and R120S/R290S) to hAOC3 was at the background level (Fig 6C, Figure C in S2 Fig)[43][44].

### Siglec-9-EC enhances the enzymatic activity of CHO-hAOC3 cells

**Fig 7:**
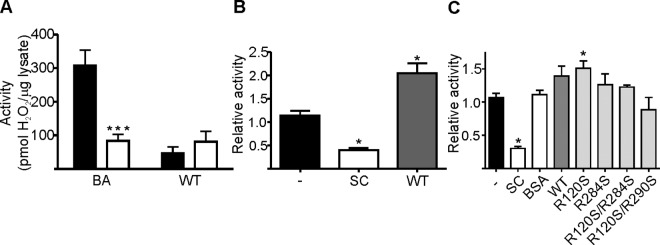
The interplay between the binding of Siglec-9 to hAOC3 and the enzymatic activity of hAOC3. **(A)** We detected no activity of CHO-hAOC3 cell lysates (black columns) on Siglec-9-EC (WT) when compared to inactive CHO-hAOC3-Y471F lysate (white columns). Typical activity of CHO-hAOC3 cells was detected towards positive control substrate benzylamine (BA) and it was significantly higher than for the control CHO-hAOC3-Y471F cells (Z = -2.72, *p* = .006). Means of six experiments ± SEM. **(B)** The relative activity of CHO-hAOC3 cells on labeled BA, when known inhibitor semicarbazide (SC) or 5 μM Siglec-9-EC (WT) was added before the substrate. Mean of three independent experiments ± SEM are shown. The effect of Siglec-9-EC on the activity of CHO-hAOC3 towards BA was significantly elevated when compared to the activity without added Siglec-9-EC (Z = -1.96, p = .05). **(C)** The relative activity of the CHO-hAOC3 cells on labeled BA, when BSA control, known inhibitor SC, 1 μM Siglec-9-EC (WT), -R120S, -R284S, -R120S/R284S or–R120S/R290S was added before the substrate. Mean of three independent experiments ± SEM are shown. SC significantly reduces the activity of hAOC3 cells (Z = -2.31, *p* = .021) and Siglec-9-EC increases the activity of hAOC3 cells, although the difference is not statistically significant (Z = -1.41, p = .157). The R120S mutant, however, does increase the activity significantly (Z = -2.12, *p* = .034) whereas the R284S mutant does not (Z = -1.06, *p* = .289). In addition, both of the double mutants (R120S/R284S: Z = -0.87, *p* = .386; R120S/R290S: Z = -1.77, *p* = .077) abolish the effect of Siglec-9-EC because they fail to increase the activity of hAOC3. *: p<0.05, **:p<0.01 and ***:p<0.001.

Next, we tested if the purified Siglec-9-EC could act as a substrate for hAOC3. We were not able to demonstrate any activity over the negative control (CHO-hAOC3-Y471F lysate, Fig 7A), when we tested the activity of CHO-hAOC3 lysate on 10 μg of Siglec-9-EC using the Amplex Red assay. However, when we analyzed the effect of Siglec-9-EC on the amine oxidase activity of intact CHO-hAOC3 cells, we monitored a two-fold increase in the benzylamine activity of CHO-hAOC3 (Fig 7B). Thereafter, we studied if the Arg/Ser mutations have an effect on the activity-modulator capacity of Siglec-9-EC (Fig 7C). This time, we saw a similar increase in the hAOC3 activity with both WT and Siglec-9-EC/R120S mutant proteins. Thus, Siglec-9-EC was able to modulate the amine oxidase activity of CHO-hAOC3 cells. Furthermore, Siglec-9-EC/R120S was still able to enhance the amine oxidase activity of hAOC3, although the R120S mutation in the V domain had disrupted the sialic acid binding capacity of Siglec-9-EC (Fig 7C) whereas the mutation of R284S in the C2_2_ domain and the double mutations (R120S/R284S and R120S/R290S) had lost the capacity to modulate the hAOC3 activity.

### Semicarbazide does not have an effect on Siglec-9-EC–hAOC3 interaction whereas imidazole inhibits the binding of Siglec-9-EC to hAOC3

**Fig 8:**
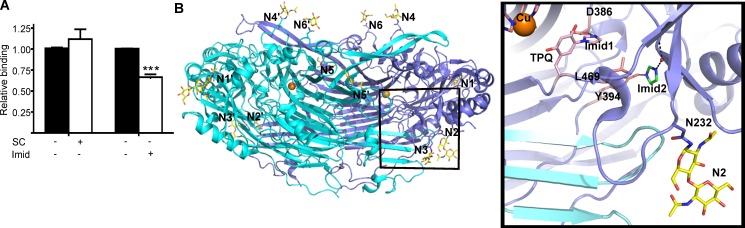
Effect of hAOC3 inhibitors on the Siglec-9-ECn the subsequent Si **(A)** Imidazole reduces Siglec-9-EC binding to hAOC3 whereas semicarbazide has no effect. Black bars: no added inhibitor, white bars: 1 mM semicarbazide (SC) or 50 mM imidazole (Imid) added. Average of two independent results are shown ± SEM. **(B)** The overall structure of heavily glycosylated hAOC3. Chain A in the hAOC3 dimer is shown in blue, chain B in cyan and the copper ions in each deeply buried active site are shown as orange spheres. The X-ray structures of hAOC3 have revealed that the 12 N-glycosylation sites in the hAOC3 dimer are glycosylated but only a few sugar units of the highly flexible N-glycans are visible in the structures. The attached glycans are named N1 (attached to N137), N2 (N232), N3 (N294), N4 (N592), N5 (N618) and N6 (N666) in chain A and those in chain B marked with an additional hyphen. Close-up view corresponds to the boxed area of the hAOC3 dimer and shows the active site channel of hAOC3 in complex with imidazoles (PDB ID 2Y74 [32]). Like semicarbazide, Imid1 (salmon) directly binds to the TPQ cofactor in the active site whereas Imid2 (green) forms a hydrogen bond with Y394 in the channel and blocks access to the active site. Similarly to Imid2, the Arg guanidinium of Siglec-9 could interact with the polar residues in the Imid2-binding site when semicarbazide (like Imid1) is covalently bound to the TPQ cofactor. The sugars of the N232-attached glycan (N2) on the surface of hAOC3 are shown as sticks.

Our earlier data on the binding of a Siglec-9 peptide suggested that Siglec-9 binds directly to the TPQ cofactor of hAOC3 [6] but the activity and inhibition assays carried out in this study challenged this assumption. Since Siglec-9 clearly modulates the enzymatic activity of hAOC3, we further tested whether the binding of semicarbazide to hAOC3 has an effect on the Siglec-9-EC–hAOC3 interaction. Semicarbazide is a classical amine oxidase inhibitor that binds covalently to the TPQ cofactor and inhibits the enzymatic activity irreversibly. Binding of 1 mM semicarbazide to hAOC3 had no effect on the subsequent Siglec-9-EC adhesion (Fig 8A, Figure D in S2 Fig).

From our previous work, we know that imidazole at high concentrations inhibits hAOC3 in a reversible manner and the X-ray structure of the hAOC3-imidazole complex shows two distinct sites in the active site cavity [36]: one of the imidazole molecules (Imid1) interacts with TPQ in the active site whereas the other one (Imid2) binds into the active site channel (Fig 8B). To elucidate if imidazole binding has an effect on the Siglec-9-EC–hAOC3 interaction, we injected several concentrations (5–100 mM) of imidazole together with the Siglec-9-EC samples over the hAOC3 surface. The addition of imidazole to the binding assay non-linearly decreased the binding of Siglec-9-EC to hAOC3 (Spearman r_s_ = -0.8, *p* = 0.05, data not shown) and at the concentration of 50 mM inhibited the binding of Siglec-9-EC to hAOC3 by about 30% (Figure D in S2 Fig), when compared to the binding without imidazole (Fig 8A).

## Discussion

Previously, we have proven by *in vitro*, *ex vivo* and *in vivo* studies that Siglec-9 interacts with hAOC3 [6] but the interaction has not earlier been characterized in detail. Our previous results with Siglec-9 derived peptides indicated that R284 and R290 in the C2_2_ domain of Siglec-9 might have a role in the protein-protein interaction [6] but the role of the V domain was not studied at all. In this study, our specific aim was to clarify, which domains in the extracellular part of Siglec-9 are important for the interaction and what is their exact role in the interaction. Towards this goal, we have produced a soluble, biologically active, extracellular domain of Siglec-9 and modeled its 3D structure, which consists of the V, C2_1_ and C2_2_ domains, and studied its interaction with hAOC3. Using both wild-type and mutant forms of recombinant Siglec-9-EC proteins, we have for the first time, to our knowledge, shown that both the C2_2_ and V domain of Siglec-9 have a specific role in its interactions with hAOC3.

Our results with the recombinant Siglec-9-EC proteins prove that Siglec-9 and hAOC3 are able to interact directly without any additional factors (Fig 5A and 5B), which was not clear from the previous cell-based assays [6]. Actually, the affinity of Siglec-9-EC to hAOC3 (*K*_*d*_ of 1.04 ± 0.87 μM) is one magnitude higher than the values measured for the previously known Siglec-glycan interactions, which are at the range of 0.1–3 mM [1, 45, 46]. We therefore wondered if the higher affinity results from the unique protein-protein interactions that mediate Siglec-9 binding to hAOC3. To our surprise and in contrast to previous results with the R284A and R290A mutants of the Siglec-9 peptides [6], the R284S and R290S mutations of Siglec-9-EC did not weaken but rather enhanced binding (Table 1). Our earlier model for Siglec-9–hAOC3 interaction [6] proposed that either R284 or R290 directly binds to the TPQ cofactor but the observed enhanced binding of the R284S and R290S mutants rules out this possibility since a serine cannot bind directly to TPQ. Therefore, the interaction between the recombinant Siglec-9-EC and hAOC3 cannot result from the specific binding of R284 or R290 to TPQ.

We next tested if the strong binding of Siglec-9 to hAOC3 was due to the well-known sialic acid binding ability of the Siglec-9 V domain [16]. Because addition of free sialic acids interfered with the Siglec-9-EC-hAOC3 interaction (Fig 6A) and the removal of the sugar-binding V domain or the sugar-binding residue R120 abolished the binding almost completely (Fig 6C), we can conclude that, especially under flow conditions, the binding of Siglec-9-EC to hAOC3 is *mainly* mediated via the V domain.

R284 and R290 in C2_2_ are clearly involved in binding Siglec-9 to hAOC3 but where do they bind? Siglec-9-EC cannot bind to TPQ since Siglec-9-EC was not a substrate or inhibitor for hAOC3 (Fig 7A and 7B), the TPQ-binding semicarbazide inhibitor did not block the Siglec-9-EC–hAOC3 interaction (Fig 8A) and the R284S mutation increased the binding affinity of Siglec-9-EC (Table 1). However, imidazole significantly impaired the interaction (Fig 8A) and, therefore, the binding site for Siglec-9-EC plausibly overlaps with the secondary imidazole-binding site (Imid2, Fig 8B) in the active site channel of hAOC3 [36]. This site has many polar residues (e.g. Y394 in Fig 8B), which may form hydrogen bonds with R284 and R290 in Siglec-9-EC. Similarly, the hydroxyl group of serines in Siglec-9-EC/R284S and Siglec-9-EC/R290S is capable of forming hydrogen bonds with these polar residues. The fact that side chain of a serine is smaller and fits better into the active site cavity of hAOC3 than the large and bulky arginine in the Siglec-9-EC might explain the improved binding properties of the R284S and R290S mutants compared to the WT. It is intriguing that the reversible pyridazinone inhibitors of hAOC3 activity also bind to this unique binding site [47], which now seems to be a physiological binding site as well.

Could Siglec-9 affect the activity of hAOC3 by binding to the active site channel? Siglec-9-EC is neither a substrate (Fig 7A) nor an inhibitor (Fig 7B). To our surprise, however, Siglec-9-EC *increased* the benzylamine activity of hAOC3 about two-fold in the cell-based assay (Fig 7B). Furthermore, the R120S mutant, almost incapable of binding to hAOC3 under flow conditions (Fig 6C), modulated the amine oxidase activity like the WT protein (Fig 7C), but the R120S/R284S and R120S/R290S double mutants had lost this capacity (Fig 7C). This result explains our previous data according to which the removal of six N-glycosylation sites from the hAOC3 dimer could simultaneously reduce lymphocyte binding and increase enzymatic activity [14]. Consequently, in this study we have discovered a biological role for the binding of R284 and R290 to the active site of hAOC3: upon binding they modulate the amine oxidase activity of hAOC3.

The molecular dynamics simulations of the 3D model for Siglec-9-EC revealed its flexibility and support the idea of conformational changes in the 3D arrangement of V-C2_1_ and C2_2_ upon hAOC3 binding. Furthermore, the recent analysis of small-angle X-ray scattering structures of *Lens esculenta* and *Euphorbia characias* amine oxidases [48] showed that the D3 domain of the copper amine oxidase fold makes a rigid-body movement and opens up the buried active site to make it more easily accessible for ligands. In the case of hAOC3, this is a fascinating scenario since its interaction with Siglec-9 would be enhanced if a similar movement of D3 opens the hAOC3 structure. The structural rearrangements would also give an explanation for the mechanism of Siglec-9-induced increase in the enzymatic activity of hAOC3. Due to the complex nature of the interactions and the involvement of sialic acids end groups of the highly flexible hAOC3 glycans (12 N-glycosylation sites in Fig 8B), computational predictions of the 3D complex are challenging and further experimental studies to find out e.g. which the N-glycan(s) in hAOC3 are important for the interaction are in progress. Furthermore, we cannot rule out the possibility that the glycosylation of Siglec-9 might contribute to its physiological interaction with hAOC3. Since Siglec-9-EC produced in insect cells exhibits a non-physiological glycosylation pattern, this could not be studied.

The activity-modulating effect of Siglec-9 at the cell level is an important discovery. Salmi et al. [10] have earlier shown that incubation of hAOC3 with specific antibodies enhanced the enzymatic activity towards a hAOC3 substrate on lymphocytes but decreased firm adhesion and rolling. Additionally, incubation of the endothelial cells with inhibitors also decreased the rolling and firm adhesion [10]. More interestingly, inhibitors diminished the rolling, adhesion and transmigration of granulocytes [10, 12], albeit the enzymatically inactive hAOC3-Y471F was still able to mediate the rolling of granulocytes [12]. It can be envisioned that Siglec-9 by enhancing the activity of hAOC3 modulates the hAOC3-mediated leukocyte trafficking and thus the outcome of the immune response at sites of inflammation.

It has earlier been shown that Siglec-9 is an immunosuppressive molecule [3]. Previously, Siglec-9 was reported to induce both apoptotic and non-apoptotic cell death of neutrophils [49]. The Siglec-9-mediated non-apoptotic cell death was caspase-independent but dependent on reactive oxygen species. Moreover, it occurred under *in vivo* inflammatory conditions and was characterized by cytoplasmic vacuolization [49]. Coupled to our results, this suggests that hAOC3 could be the previously unknown ligand for Siglec-9 in non-apoptotic cell death since the Siglec-9-mediated enhanced enzymatic activity of hAOC3 produces elevated levels of hydrogen peroxide and thus increases the reactive oxygen species at the sites of inflammation. This function of Siglecs is highly cell-type specific since Siglec-7, unlike Siglec-9, induced the non-apoptotic cell death of the U937 cells [50]. Strikingly, the CE-loop of C2_2_ in Siglec-7 was crucial for the Siglec-7-mediated non-apoptotic cell death [50]. The cell death activity induced by the extracellular part of Siglec-7 was significantly decreased when any of the key residues (W288, T289 and S292) in the CE-loop of Siglec-7 was replaced by the corresponding residue in Siglec-9 (L287, S288 and G291) [50]. Interestingly, L287 is exclusively found in Siglec-9 and replaced by a tryptophan in the C2 domains of the other CD-33-related Siglec sequences (Fig 1B). R284 and R290 of Siglec-9, which are located in the vicinity of theses residues, are conserved only in the C2_3_ domain of Siglec-10 and the C2_2_ domain of Siglec-7 and (Fig 1B) but their importance for Siglec-7 function is unknown.

Although further studies are needed to elucidate the biological implications of Siglec-9–hAOC3 interactions, it is tempting to speculate how they might mediate the different steps in the extravasation cascade: 1) The rolling of leukocytes could be mediated via the interactions between the V domain of Siglec-9 and the sialic acids on hAOC3; 2) The firm adhesion might also require contacts from the C2_2_ domain of Siglec-9; and 3) The transmigration might be mediated by the enzymatic activity of hAOC3, which is enhanced by the interaction of Siglec-9 with the active site channel of hAOC3.

## Conclusions

As a conclusion, our results prove that the Siglec-9–hAOC3 interaction is multivalent and much more complex than expected. We interpret our findings so that the interaction of Siglec-9-EC with hAOC3 is mediated both by *protein-sugar interactions via the V domain* and *by the protein-protein interactions via the C2*_*2*_ *domain*. This is the first time, to our knowledge, when both the C2_2_ and V domain of Siglec-9 are proved to be associated with its interactions with any ligand. We could also postulate that R284 and R290 in C2_2_ interact with hAOC3 in a manner that increases the amine oxidase activity of hAOC3, which is known to be important for hAOC3-mediated leukocyte trafficking [12].

## Supporting Information

S1 Fig The quality assessment of the homology models. The graphs present the quality assessment of the modeled structures by ProSAWeb (Sippl, 1993; Wiederstein and Sippl) and QMEAN Benkert et al., 2009. The ProSAWeb scores for V-C2_1_ **(A)** and **(B)** C2_2_ (shown as a black dot) are within the scores of the experimentally solved structures of similar size. The QMEAN Z-scores for V-C2_1_ **(C)** and C2_2_ **(D**) (shown as a red cross) are similar to the Z-scores of the PDB structures of the same size in the reference set.
(DOCX)

S2 Fig Representative sensorgrams of the surface plasmon resonance binding experiments **(A**) Determination of the binding constants for 1 μM Siglec-9-EC, Siglec-9-EC/R284S and Siglec-9-EC/R290S. Each curve was used separately to determine *k*_*on*_ and *k*_*off*_, which were used for the *K*_*D*_ determination. (**B**) The binding of 0.5 μM Siglec-9-EC with and without different concentrations with sialic acid or with disialyl lactotetraosylceramide (DSLc4) to immobilized hAOC3. For both, one out of 2 experiments are shown. In the first experiment, the curve for the binding of Siglec-9-EC with 0.5 μM displayed substantial noise at the end of injection, most probably due to the air bubble. For this curve, the relative binding was determined before the noise. (**C**) The binding of 0.5 μM Siglec-9-EC and the Siglec-9-EC mutants to immobilized hAOC3. One out of 2–3 experiments are shown. (**D**) The binding of 0.5 μM Siglec-9-EC with and without irreversible inhibitor (1 mM semicarbazide, SC) or reversible inhibitor (50 mM imidazole) to immobilized hAOC3. The binding of Siglec-9-EC is similar before and after SC. One out of 2 experiments are shown.
(DOCX)

S1 Text The cloning procedure of the Siglec-9 gene (DOCX)
