# Supplementary material for: Multivalent Interactions of Human Primary Amine Oxidase with the V and C22 Domains of Sialic Acid-Binding Immunoglobulin-Like Lectin-9 Regulate Its Binding and Amine Oxidase Activity
Source: PLoS One. 2016 Nov 28;11(11):e0166935. doi: 10.1371/journal.pone.0166935 (PMC5125647; doi:10.1371/journal.pone.0166935)
Supplement: S1 Fig — The graphs present the quality assessment of the modeled structures by ProSAWeb (Sippl, 1993; Wiederstein and Sippl) and QMEAN Benkert et al., 2009. The ProSAWeb scores for V-C21 (A) and (B) C22 (shown as a black dot) are within the scores of the experimentally solved structures of similar size. The QMEAN Z-scores for V-C21 (C) and C22 (D) (shown as a red cross) are similar to the Z-scores of the PDB structures of the same size in the reference set. (DOCX) [file pone.0166935.s001.docx]

**S1 Figure. The quality assessment of the 3D homology models for the V-C21 domains and the C22 domain of Siglec-9**


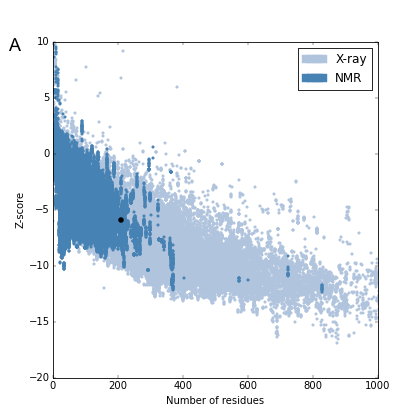

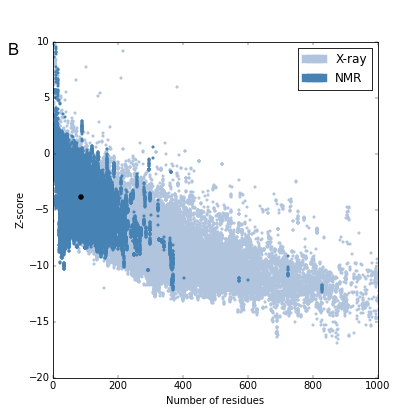


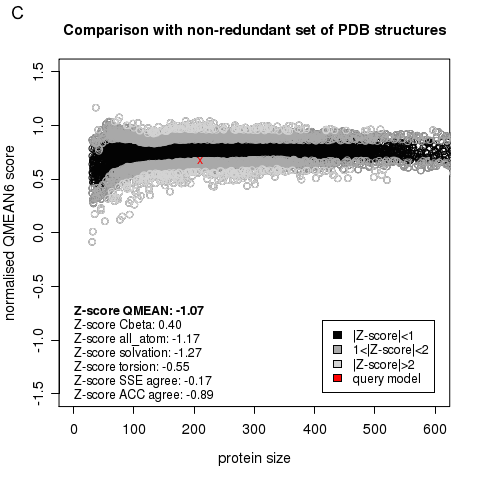

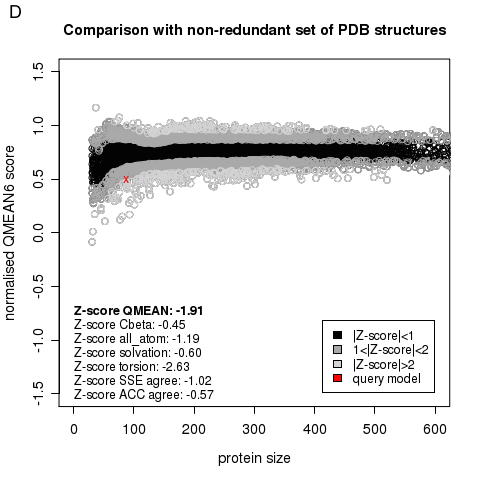


**Fig S1. Quality assessment of the homology models.** The graphs present the quality assessment of the modeled structures by ProSAWeb (Sippl, 1993; Wiederstein and Sippl) and QMEAN Benkert et al., 2009. The ProSAWeb scores for V-C2_1_ **(A)** and **(B)** C2_2_ (shown as a black dot) are within the scores of the experimentally solved structures of similar size. The QMEAN Z-scores for V-C2_1_ **(C)** and C2_2_ **(D**) (shown as a red cross) are similar to the Z-scores of the PDB structures of the same size in the reference set.
