# Supplementary material for: Multivalent Interactions of Human Primary Amine Oxidase with the V and C22 Domains of Sialic Acid-Binding Immunoglobulin-Like Lectin-9 Regulate Its Binding and Amine Oxidase Activity
Source: PLoS One. 2016 Nov 28;11(11):e0166935. doi: 10.1371/journal.pone.0166935 (PMC5125647; doi:10.1371/journal.pone.0166935)
Supplement: S2 Fig — (A) Determination of the binding constants for 1 μM Siglec-9-EC, Siglec-9-EC/R284S and Siglec-9-EC/R290S. Each curve was used separately to determine kon and koff, which were used for the KD determination. (B) The binding of 0.5 μM Siglec-9-EC with and without different concentrations with sialic acid or with disialyl lactotetraosylceramide (DSLc4) to immobilized hAOC3. For both, one out of 2 experiments are shown. In the first experiment, the curve for the binding of Siglec-9-EC with 0.5 μM displayed substantial noise at the end of injection, most probably due to the air bubble. For this curve, the relative binding was determined before the noise. (C) The binding of 0.5 μM Siglec-9-EC and the Siglec-9-EC mutants to immobilized hAOC3. One out of 2–3 experiments are shown. (D) The binding of 0.5 μM Siglec-9-EC with and without irreversible inhibitor (1 mM semicarbazide, SC) or reversible inhibitor (50 mM imidazole) to immobilized hAOC3. The binding of Siglec-9-EC is similar before and after SC. One out of 2 experiments are shown. (DOCX) [file pone.0166935.s002.docx]

**S2 Figure. Representative sensorgrams of the surface plasmon resonance binding experiments of Siglec-9 to immobilized hAOC3**

**
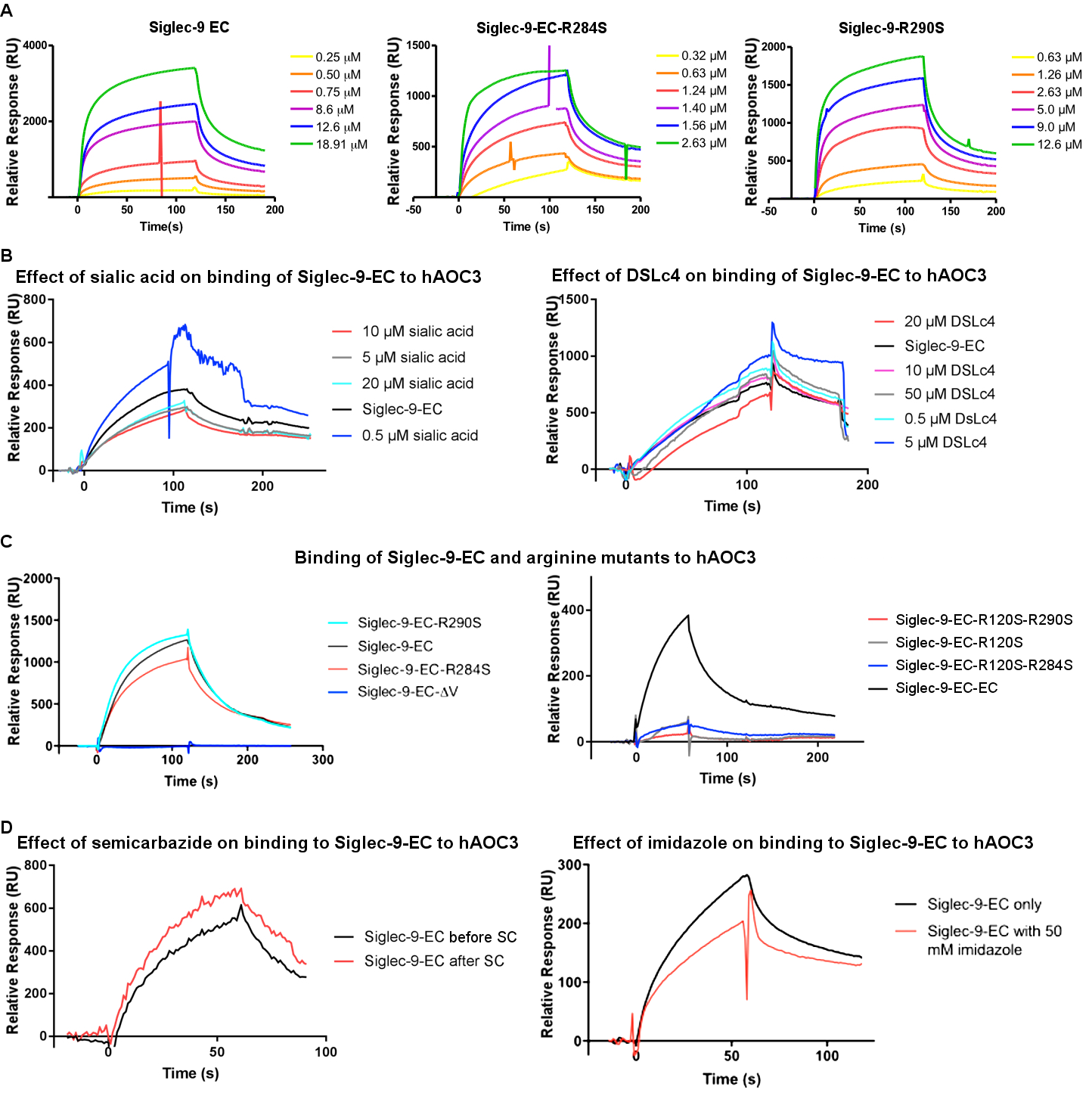
**

**Fig S2. Representative sensorgrams of the surface plasmon resonance binding experiments (A**) Determination of the binding constants for 1 μM Siglec-9-EC, Siglec-9-EC/R284S and Siglec-9-EC/R290S. Each curve was used separately to determine *k_on_* and *k_off_*, which were used for the *K_D_* determination. (**B**) The binding of 0.5 μM Siglec-9-EC with and without different concentrations with sialic acid or with disialyl lactotetraosylceramide (DSLc4) to immobilized hAOC3. For both, one out of 2 experiments are shown. In the first experiment, the curve for the binding of Siglec-9-EC with 0.5 μM displayed substantial noise at the end of injection, most probably due to the air bubble. For this curve, the relative binding was determined before the noise. (**C**) The binding of 0.5 μM Siglec-9-EC and the Siglec-9-EC mutants to immobilized hAOC3. One out of 2-3 experiments are shown. (**D**) The binding of 0.5 μM Siglec-9-EC with and without irreversible inhibitor (1 mM semicarbazide, SC) or reversible inhibitor (50 mM imidazole) to immobilized hAOC3. The binding of Siglec-9-EC is similar before and after SC. One out of 2 experiments are shown.
