# Supplementary material for: Multivalent Interactions of Human Primary Amine Oxidase with the V and C22 Domains of Sialic Acid-Binding Immunoglobulin-Like Lectin-9 Regulate Its Binding and Amine Oxidase Activity
Source: PLoS One. 2016 Nov 28;11(11):e0166935. doi: 10.1371/journal.pone.0166935 (PMC5125647; doi:10.1371/journal.pone.0166935)
Supplement: S1 Text — (DOCX) [file pone.0166935.s003.docx]

**S1 Text. The cloning procedure of the Siglec-9 gene**

The cloning of the Siglec-9 gene for the production in insect cells and mutagenesis including sequential process with the primer sequences used in each step were done according to the describe procedure.

Used template, Siglec-9 in pcDNA3, has been described earlier (Zhang JQ, Nicoll G, Jones C, Crocker PR (2000) *J Biol Chem*, 275(29):22121-6).

**Step 1.** Introducing a HindIII site (underlined) after Siglec-9 gene encoding for the extra cellular Siglec-9 and the deletion of C-terminal His-tag by polymerase chain reaction. Details of the method in the article, used primers (f for forward, r for reverse):

fHE-48 5’-TGA GAT CCG GCT GCT AAG CTT GTC-3’

rHE-49 5’-CCC CTG AGT CAC TCC TGA TGT G-3’

**Step 2.** Cloning of XbaI/HindIII fragment (Siglec-9-EC and some extra bases from a vector) to p503.9 resulting to pHE-39 with Siglec-9-EC without His-tag.

**Step 3.** Insertion of a cleavable N-terminal His-tag (rTEV site in bold) and a production of different deletion mutants of Sigec-9:

for Siglec-9-EC (V-C2_1_-C2_2_; encoding plasmid pHE-40):

rHE-50 5’-CTT GTC ATC GTC GTC CTT GTA GTC TG-3’

fHE-51 5’- CACCACCACCACCACCAC **GAA AAT CTT TAT TTT CAA TCA**

ATGATGCAGAGTTCCGTGACGG-3’

for Siglec-9 without V-domain (C2_1_-C2_2_; pHE-41)

rHE-50 (above)

fHE-52 5’-CACCACCACCACCACCAC **GAA AAT CTT TAT TTT CAA TCA +** CCCAACATCCTCATCCCAGG-3’

for Siglec-9-C2_2_ (pHE-42)

rHE-50 (above)

fHE-53 5’-CACCACCACCACCACCAC **GAA AAT CTT TAT TTT CAA TCA**

CCTCAGAACTTGACCATGACTGTCT -5’

**Step 4**. Introducing mutations to Siglec-9 (pHE-40 used as a template)

for Siglec-9-EC-R290S mutant (resulting plasmid pHE-46):

fHE-56 5'-CCCCCTGCCAGCCTGAGCCTGAG-3'

rHE-57 5'-CTCAGGCTCAGGCTGGCAGGGGG-3'

for Siglec-9-EC-R284S mutant (pHE-47):

fHE-58 5'-CCTGAGCTGGAGCGGCCTGACCCTG-3'

rHE-59 5'-CAGGGTCAGGCCGCTCCAGCTCAGG-3'

for Siglec-9-EC-R120S, Siglec-9-R284S/R120S and Siglec-9-R290S/R120S mutants (pHE-48, pHE-49 and pHE-50, respectively):

fHE-60 5'-GCGGGGAGATACTTCTTTAGTATGGAGAAAGGAAGTA-3'

rHE-61 5'-TACTTCCTTTCTCCATACTAAAGAAGTATCTCCCCGC-3'
